# Nutrient variation induced by rodent disturbance in *Haloxylon ammodendron* as a target transfer strategy

**DOI:** 10.1002/ece3.8362

**Published:** 2021-11-25

**Authors:** Wenqin Zhao, Hanli Dang, Tao Zhang, Jianrui Dong, Hongwei Chen, Wenjie Xiang

**Affiliations:** ^1^ College of Life Sciences Shihezi University Shihezi City China; ^2^ Xinjiang Production and Construction Corps Key Laboratory of Oasis Town and Mountain‐basin System Ecology Shihezi City China; ^3^ Key Laboratory of Oasis Eco‐agriculture College of Agriculture Shihezi University Shihezi City China

**Keywords:** Gurbantunggut Desert, *Haloxylon ammodendron*, nutrient variation, rodent disturbance

## Abstract

Nutrients form a link between herbivores and plant. This study explored the physiological and ecological response mechanism of *Haloxylon ammodendron* population to rodent disturbance in Gurbantunggut Desert from the perspective of nutrient cycle. Through field investigation, we quantified rodent disturbance intensity (DI) to *H. ammodendron* and analyzed the ecological response mechanism of *H. ammodendron* population to rodent disturbance from the perspective of plant and soil nutrient cycling and changes. The results indicated that moderate rodent DI (number of effective burrows = 3–6) was the maximum limit that can be tolerated by *H. ammodendron*; the threshold for optimal *H. ammodendron* response to rodent disturbance was mild (number of burrows = 1–3). Meanwhile, the rodent disturbance caused significant nutrient enrichment (e.g., organic carbon, available phosphorus, and available potassium) in the deeper soil (at 20–40 and 40–60 cm depth) and significantly reduced the soil total salt content (*p* < .05). Furthermore, as the DI increased, the branches of *H. ammodendron* showed significantly increased soluble total sugar, crude fiber, and total nitrogen contents (*p* < .05) but significantly decreased crude fat and crude protein contents (*p* < .05); these results are related to the nutritional target transfer strategy evolved by *H. ammodendron* for long‐term resistance to rodent disturbance. The current study clarified the optimal disturbance model for mutually beneficial *H. ammodendron*–great gerbil relationship, on the basis of which the ecological response mechanism of *H. ammodendron* population to rodent disturbance in deserts was illustrated. The current study provides a scientific basis for the protection mechanisms of desert plants to rodent disturbance.

## Graphical abstract

To decipher the plant and herbivore interaction in the Gurbantunggut Desert, this study quantified the disturbance level of rodents to *Haloxylon ammodendron* and analyzed the ecological response mechanism of *H. ammodendron* population to rodent disturbance from the perspective of plant and soil nutrient cycling and changes. Our study showed that gerbil disturbance caused significant nutrient enrichment in the deeper soil (20–40 and 40–60 cm) (e.g., organic carbon, available phosphorus, and available potassium), but significantly reduced the total salt content in the soil (*p* < .05). Furthermore, with the increase of disturbance intensity, the contents of soluble total sugar, crude fiber, and total nitrogen in assimilated branches of *H. ammodendron* were significantly increased, while the contents of crude lipid and crude protein were significantly decreased (*p* < .05), which was related to the nutritional target transfer strategy evolved by *H. ammodendron* during the long‐term resistance to rodent disturbance.

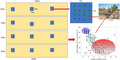

## 1 INTRODUCTION

Plant–herbivore relationships are complex and involve interactions such as complex molecular, signaling, and strategy networks aimed at overcoming each other's defenses. Herbivores use various feeding strategies to obtain nutrients from plants. Plants, in turn, protect themselves by triggering defense mechanisms that suppress, block, or modify the metabolism of herbivores (Santamaria et al., 2013; Xiao et al., 2017). In response to the constant threat of herbivorous animals, plants have evolved a range of defense strategies. These include mechanical and chemical barriers that reduce the performance of herbivores; for instance, after a herbivore attacks a plant, it usually releases a wide variety of herbivore‐induced plant volatiles (HIPVs) that can attract the herbivore's natural enemies and warn the neighboring plants of an imminent threat. Exposure to HIPVs increases the levels of defense signal hormones in plants, changes their defense status, and makes the undamaged branches more resistant to herbivores; moreover, surrounding plants may respond to HIPV signals by assessing the risks and producing plasticity response based on the actual environmental conditions (Pezzola et al., 2017; Rodriguez‐Saona & Frost, 2010).

Of the many defense strategies, the mobile target strategy may be a robust response in an unpredictable and information‐free environment (Kessler, 2015). Plants using the defense strategy of moving targets respond to attacks by changing their phenotypes, or even their genotypes. Moreover, considering the plastic performance of the defense strategies, these plants invest resources in defense only when the risk of damage increases (representing a cost–benefit trade‐off), thereby avoiding the waste of resources (Potts, 2021). The cost of complementary utilization of resources due to changes in plant genotypes is often manifested in the nutritional level; this increases resistance to herbivores (Mcart & Thaler, 2013; Velzen & Etienne, 2015), indicating a certain relationship of plant nutrient content and community structure with the number of herbivores.

This relationship has been a concern of ecologists for a long time. For instance, Awmack and Leather (2001) found that the relationship of plant nutrition with the growth and survival of herbivore population is always concave, and the difference in nutrient composition greatly affects the fecundity of the herbivore population. Moreover, Wetzel et al. (2016) found that plants inhibit herbivorous population via changes in plant nutrient levels; this is a key herbivore inhibition mechanism in natural systems. Joern et al. (2012) confirmed that nitrogen and phosphorus content is positively correlated with the number of herbivores and that other nutrients may have a role here. Herbivores require various nutrients; however, most researchers have focused only on nitrogen (i.e., plant protein) and phosphorus contents and paid little attention to other nutrients. Moreover, most relevant reports have focused on insect–plant relationships and limited empirical studies on the relationship between plant nutrient content and community structure of herbivorous in desert ecosystems (Awmack & Leather, 2001; Scogings, 2011; Vieira‐Neto et al., 2017; Wetzel et al., 2016; Wimp et al., 2010).

In desert ecosystems, rodents always live alongside desert plants. These rodents obtain the nutrients they need for their growth and development by feeding on the plants. Therefore, the nutrient types and contents in the plant tissues have a considerable effect on the rodents’ growth and development. *Haloxylon ammodendron*, representing the most widely distributed vegetation in desert areas, plays an important ecological role in maintaining the stability of sand dunes, providing essential nutrient and water conditions for other vegetation demonstrating undergrowth, and maintaining arid ecosystem function and structure (Mares et al., 1997; Zhao et al., 2019). The great gerbil (*Rhombomys opimus*)—a main, highly social rodent in the desert and semidesert areas of Northwest China—is widely distributed in *H. ammodendron* forests (Wen et al., 2020). It feeds on desert plants including not only *H. ammodendron* but also *Kalidium foliatum*, *Tamarix taklamakanensis*, *Caragana sinic*a, and other psammophytes. Among them, the nutritious branches of *H. ammodendron* account for 80% of its food source (Liu et al., 2012). *Rhombomys opimus* burrows and nests under *H. ammodendron* shrubs and gnaws on their root phloem (Yang et al., 2011); this activity has affected the growth of *H. ammodendron* forests in the Gurbantunggut Desert of Xinjiang, China (Gao et al., 2014).

In deserts with a relatively homogeneous population structure, *R. opimus* and *H. ammodendron* demonstrate long‐term coexistence. *Rhombomys opimus* mainly harm the branches in 2–3 years *H. ammodendron*, and only high intensity disturbance can affect the growth of *H. ammodendron*, while light feeding can increase branches number and crown width, which was beneficial to the growth of *H. ammodendron*. As a consequence of long‐term feeding and disturbance by rodents, *H. ammodendron* has gradually evolved a unique evolutionary strategy to ensure its own survival. Simultaneously, to keep its food source, *R. opimus* has gradually evolved a corresponding survival mechanism to maintain a balanced relationship. So far, however, no reports have been published on this topic.

Therefore, this study investigated the changes in the nutrient composition of *H. ammodendron* and soil under different *R. opimus* disturbance intensities (DIs; including no, mild, moderate, and severe). We hypothesize that *R. opimus* disturbance influences desert plant growth and that the plasticity of *H. ammodendron* nutrient composition is involved in *R. opimus* population disturbance, which triggers the moving target defense strategy in the plant. Here, the research objects were both the soil under and the branches of *H. ammodendron* exposed to different degrees of *R. opimus* disturbance in the Gurbantunggut Desert. The aims of this study were to (1) determine the critical threshold for the maximum reciprocal relationship between *H. ammodendron* and rodents through the relationship between the number of rodent burrows under *H. ammodendron* shrubs and rodent disturbance intensities (DIs), (2) elucidate the corresponding changes in *H. ammodendron* and soil nutrient compositions as a result of *R*. *opimus* disturbance, and (3) identify the optimal disturbance mode that maximizes the mutually beneficial relationship between *H. ammodendron* and *R. opimus* in the Gurbantunggut Desert.

## 2 MATERIALS AND METHODS

### 2.1 Experimental site

The field experiment was conducted at the southern edge of the Gurbantunggut Desert (44°26′–45°12′N, 86.2°06′–87°54′E) at an altitude of 314–436.8 m above sea level. This region has a typical temperate arid desert climate with an annual average temperature of 5–6.1°C; the maximum temperature can reach 43°C in the summer, and the minimum temperature can be less than −42°C in the winter. The annual cumulative temperature is as high as 3000–3600°C, and the mean annual evaporation is >2000 mm. This region is extremely dry, with an average relative humidity of 50–60%; and <45% during May and August (Huang et al., 2015). The annual precipitation is 100–160 mm, mainly occurring in the winter and during May–September and accounting for 70–80% of the annual precipitation. The stable snow cover, with a thickness of >13 cm in the winter, is an important source of deep water for vegetation growth and development in the spring (Xing et al., 2013). The landform in the region is mainly composed of fixed and semifixed dunes, sand ridges, composite dunes, and sandy interdune lowlands. This region has very few vegetation species, with *H. ammodendron* and *Haloxylon persicum* being the main species providing an approximately 20–30% of vegetation cover (Huang et al., 2015). This region has other plant species that are sporadically distributed—ranging from shrubs such as *Tamarix* spp., *Calligonum* sp., *Ephedra distachya*, *Artemisia desertorum*, and *Nitraria sibirica* to herbs species such as *Alhagi sparsifolia*, *Salsola* spp., *Aristida peuuata*, and *Suaeda* spp. (Kotler et al., 2002).

Human activity and global climate change have caused the natural vegetation in the desert to degrade seriously, leading to significant decrease in vegetation cover. In some areas, the vegetation cover has decreased to <5% (Ding et al., 2017). Moreover, the decline in the vegetation cover has severely restricted the food sources of desert rodents. Therefore, *H. ammodendron*—a high‐quality sand‐fixing plant that is the main food source for *R. opimus*—has inevitably become seriously disturbed, especially in the middle and lower parts of sand dunes and the interdune areas, where there are clusters of *R. opimus* burrows. As a result, collapsed areas are widespread here. In the densely *R. opimus* burrow areas, *H. ammodendron* has shown an irreversible decline and desertification. In some areas, although there are many *R. opimus* burrows, *H. ammodendron* is flourishing, especially with the dark green and highly vital collected branches.

### 2.2 Experimental design and sample collection

#### 2.2.1 Sample plots set

In June 2019, four sample plots (1000 × 200 m^2^) were set up at intervals of >1000 m. Three 50 × 50 m^2^ quadrats were set up in each sample plot for different disturbance levels along the direction of a longitudinal dune. The morphological characteristics (i.e., plant height, crown width, basal stem, and health status) of all *H. ammodendron* in every quadrat were recorded.

**FIGURE 1:**
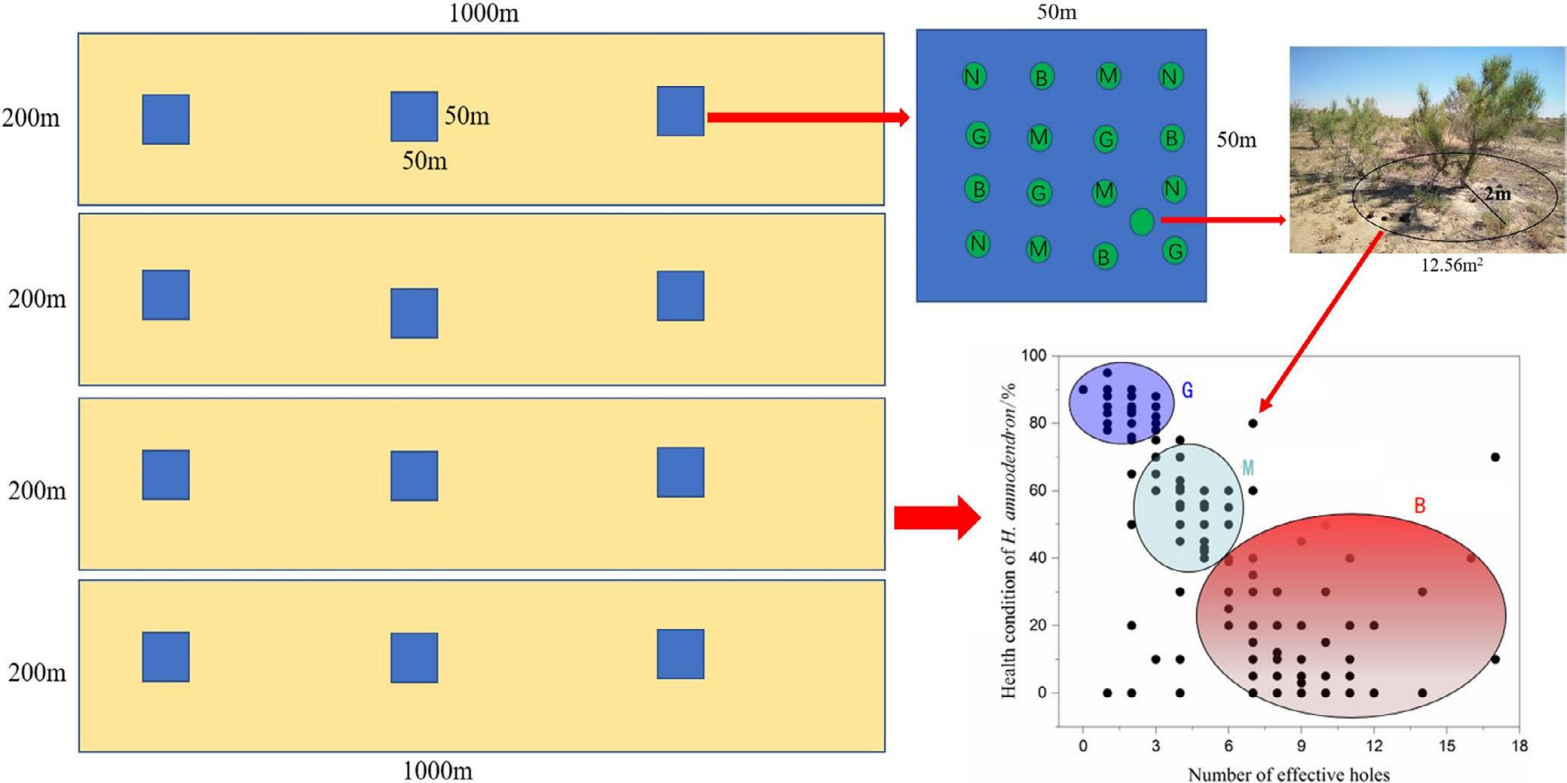
Field test design sketch. Description: Ordinate is the health condition of *Haloxylon ammodendron*; abscissa is the number of effective burrows. N, G, M, and B are, respectively, no disturbance (the number of effective burrows was 0), mild disturbance (the number of effective burrows was 1–3), moderate disturbance (3–6 effective burrows), and severe disturbance (the effective number of burrows was >6)

Each subquadrat from the 50 × 50 m^2^ quadrats in each sample plot was small and circular, measuring (2 × 2 × 3.14=) 12.56 m^2^, with *H. ammodendron* in the center were used to assess the levels of *R. opimus* disturbance (Figure 1), and 16 subquadrats were collected in each quadrat (four repetitions for each of the four different disturbance levels). In total, 192 (16 subquadrats per quadrats × 3 quadrats × 4 sample plots) subquadrats with different levels of *R. opimus* disturbance were created. At the same time, *H. ammodendron* plant areas not disturbed by *R. opimus* in the same quadrats were selected, and 108 plants (with somewhat consistent morphological characteristics) were selected according to the plant health status.

#### 2.2.2 Evaluation of the *R. opimus* effective burrow number

The *R. opimus* burrows under *H. ammodendron* shrub were randomly selected, and circular quadrats with a radius of 4 m and *H. ammodendron* plants in the center were created. Three replicates were set in each quadrat, and these quadrats are only used to evaluate the number of effective burrows. The stealing opening burrow method was adopted (Zhao et al., 2007): All *R. opimus* burrows in the sample were slightly blocked with sand, and after 24 h, the numbers of *R. opimus* burrows (i.e., the number of the reopened burrows) were recorded and considered the effective burrow numbers.

#### 2.2.3 Evaluation of *R. opimus* Dis

According to the relationship between the health status of *H. ammodendron* (percentage of healthy branches) and the *R. opimus* burrows under the bush (Figure 1), the *R. opimus* disturbance intensity (DI) was rated at four levels.

N: No *R. opimus* burrows under the crown and normal growth of *H. ammodendron* (i.e., effective burrow number = 0)
G: *R. opimus* burrows present under the crown, with obvious damage by *R. opimus* via eating twigs or burrowing and *H. ammodendron* growth somewhat affected, but >80% branches healthy (i.e., mild DI; effective burrow number = 1–3)
M: 40–80% branches healthy (i.e., moderate DI; effective burrow number = 3–6)
B: <40% branches healthy (i.e., severe DI; effective burrow number > 6).

#### 2.2.4 Evaluation of *H. ammodendron* health status in non‐*R. opimus* burrow areas

*H. ammodendron* health status in the non‐*R. opimus* burrow areas was evaluated at three levels.

NG: Normal growth (>80% healthy branches)
NM: Medium growth (40–80% healthy branches)
NB: Poor growth (<40% healthy branches)

#### 2.2.5 Plant sample collection

Five 2‐ to 3‐m‐high *H. ammodendron* plants were randomly selected from each quadrat with or without *R. opimus* disturbance, and their branches at the same height in the four cardinal directions (i.e., south, west, and north) were collected and mixed thoroughly. They were placed in Kraft paper bags and transported to the laboratory.

The branch samples were dried to a constant weight at 60°C, ground into powder using a mortar and pestle, passed through a sieve with 40 mm mesh size, and then subjected to nutritional component analysis. As described by Hansen and Koroleff (2007), crude fat (CFT) and crude fiber (CF) contents were determined using Soxhlet extraction and the acid–base decooking method (Feng et al., 2015), respectively. As described by Xiang et al. (2020), inverted and reduced sugar contents (RS), with glucose as the standard, both were determined using GB/5009.8‐2016 and GB/5009.7‐2016, respectively. Total nitrogen (TNC) content was determined using the perchloric acid–sulfuric acid digestion method. Total phosphorus (TPC) and total calcium (Ca) contents were determined through atomic absorption spectrophotometry (Type 932GBC; Scientific Equipment). As described by Li and Li (2000), soluble sugar (STS) content was determined using the anthranone colorimetric method. Soluble protein content was determined using the Coomassie bright blue method. Finally, the methods of Li were used to determine proline (Pro) content (Li & Li, 2000).

#### 2.2.6 Soil sample collection

Next, *H. ammodendron* leaves were collected, and exfoliation and separation methods were employed to collect the rhizosphere soil of *H. ammodendron*. We collected all the soil attached to the *H. ammodendron* roots. In brief, the soil loosely attached to the root system was removed by shaking several times and the remaining soil more closely attached to the root system (about 2 mm in thickness), termed rhizosphere (R) soil (Riley & Barber, 1970; Zhang et al., 2019), was then collected. At the same time, the soil in which *H. ammodendron* was not grown was selected from the same quadrats and termed nonrhizosphere (S) soil. Both R and S soils were obtained from three soil depths: 0–20, 20–40, and 40–60 cm. All soil samples were air‐dried and sieved through a 2‐mm mesh for physicochemical analysis according to Bao (2008). Soil total organic carbon (TOC), TN, TP, total potassium (TK), soil water, available nitrogen (AN), available phosphorus (AP), and available potassium (AK) contents were determined using the KCr_2_O_7_ method, HClO_4_–H_2_SO_4_ digestion method, Mo–Sb colorimetric method, atomic absorption spectrometry method, weighing method, alkaline hydrolysis diffusion method, atomic absorption spectrometry–based ammonium acetate extraction method, and 0.5 mol/L sodium bicarbonate–molybdenum blue colorimetry method, respectively. Soil pH in a 1:5 soil–deionized water mixture was measured on a pH meter (Seven Easy, Mettler‐Toledo). Rhizosphere nutrient enrichment rate (E) was calculated using the following formula (2015):

$$E=\left[\left(\text{R content}‐\text{S content}\right)/\text{S content}\right]\times 100\%$$

### 2.3 Data statistics and analysis

The differences in plant nutrients and soil physicochemical properties among different *R. opimus* DIs and soil depths were analyzed using multifactor analysis of variance on SPSS (version 19.0; IBM). Bonferroni's test was used for multiple comparisons at difference levels with analyzed using the Student–Newman–Keuls test and least significant difference (with *p* < .01 and *p* < .05 demonstrating significance, respectively). Pearson correlation analysis was performed on SPSS for soil physicochemical properties and *H. ammodendron* leaf nutrients, with the significance level set to 0.05. Redundancy analysis (RDA) of the relationship among plant nutrients, soil physicochemical properties, and *H. ammodendron* variables and the relevant mapping were performed using R (version 2.15.3).

## 3 RESULTS AND ANALYSIS

### 3.1 Effects of *R. opimus* disturbance on soil nutrients in R and S soil

**FIGURE 2:**
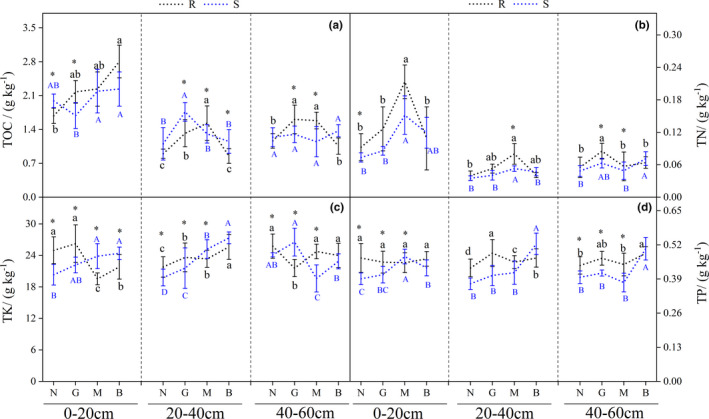
Effects of different disturbance intensity on TOC, TN, TK, and TP in the rhizosphere and nonrhizosphere soil of *Haloxylon ammodendron*. Description: Line chart (mean with standard error) with different lowercase letters indicated Rhizosphere soil (R) nutrients significant difference (*p* < .05); line chart (mean with standard error) with different capital letters indicated non‐rhizosphere soil (S) nutrients significant difference (*p* < .05); * represents significant differences between rhizosphere and nonrhizosphere nutrients at same disturbance level (*p* < .05). Ordinate is the content of soil organic carbon (TOC) (A), soil total nitrogen (TN) (B), total potassium (TK) (C), and soil total phosphorus (TP) (D); abscissa is group name (N, G, M, and B are, respectively, no disturbance (the number of effective burrows was 0), mild disturbance (the number of effective burrows was 1–3), moderate disturbance (3–6 effective burrows), and severe disturbance (the effective number of burrows was >6)). R and S were rhizosphere and nonrhizosphere soil nutrients of *H. ammodendron*, respectively

As shown in Figure 2, *R. opimus* disturbance had significant effects on R and S soil nutrients, but these effects differed at different soil depths (*p* < .05). In Figure 2A, soil TOC under G was higher in R than S soil at 0–20 cm and 40–60 cm but not 20–40 cm. In addition, soil TOC under G and M was significantly higher than under N and B in R soil at 20–40 cm and 40–60 cm but not 0–20 cm (*p* < .05) (Figure 2A). At 0–40 cm, TN content was significantly higher under M disturbance than under other DIs (*p* < .05; Figure 2B).

Under N, G, and M, TK content differed significantly between R and S soils (*p* < .05; Figure 2C). In R soil, TP content was not affected by *R. opimus* disturbance at 0–20 cm (*p* > .05) but was significantly affected by *R. opimus* disturbance at 20–40 cm (*p* < .05). In S soil, TP content at 20–60 cm was significantly higher under B than under in other samples (*p* < .05; Figure 2D).

**FIGURE 3:**
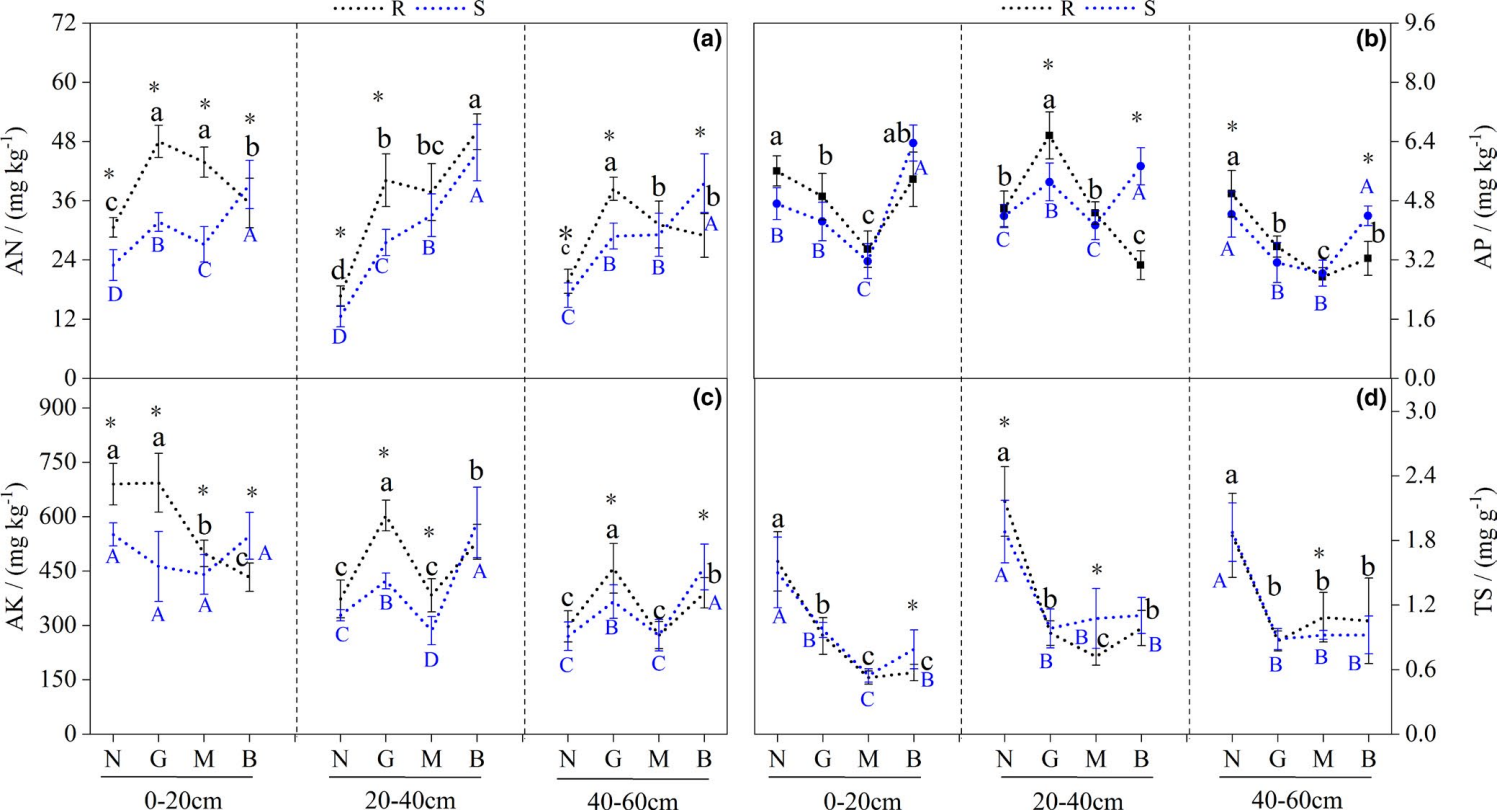
Effects of different disturbance intensity on AN, AP, AK, and TS in the rhizosphere and nonrhizosphere soil of *Haloxylon ammodendron*. Description: Line chart (mean with standard error) with different lowercase letters indicated Rhizosphere soil (R) nutrients significant difference (*p* < .05); line chart (mean with standard error) with different capital letters indicated nonrhizosphere soil (S) nutrients significant difference (*p* < .05); * represents significant differences between rhizosphere and nonrhizosphere nutrients at same disturbance level (*p* < .05). Ordinate is the content of available nitrogen (AN) (A), available phosphorus (AP) (B), available potassium (AK) (C), and total salt (TS) (D); abscissa is the group name that has identical meanings as described in Figure 2. R and S were rhizosphere and nonrhizosphere soil nutrients of *H. ammodendron*, respectively

Moreover, AN content significantly differed under N and G in both R and S soils (*p* < .05). AN content was significantly higher at 0–20 and 40–60 cm in R soil under G than in other samples (*p* < .05). AN and AP contents in S soil under B were significantly higher (*p* < .05; Figure 3A,B). AK contents in R soil were significantly higher under G (*p* < .05), whereas it was significantly higher in S soil under B (*p* < .05; Figure 3C). As *R. opimus* DI increased, total sugar (TS) content decreased significantly (*p* < .05; Figure 3D).

Finally, rhizosphere E demonstrated that under G, TN, TP, AN, AP, and AK were enriched but TS was depleted. Moreover, under M, TOC, TN, and AN were enriched, whereas TN, AP, and AK were depleted under B (Table 1).

**TABLE 1:** Enrichment ratio in rhizosphere soil of *Haloxylon ammodendron* under different disturbance intensity (DI)

Soil depth	DI	TOC	TN	TP	TK	AN	AP	AK	TS
0–20	N	−15.53	25.64	20.24	22.84	33.45	18.75	25.15	6.92
	**G**	**29.06**	**47.24**	**11.82**	**18.26**	**51.55**	**15.77**	**49.82**	**−5.78**
	M	2.11	40.56	−4.98	−18.17	61.29	10.20	13.15	−3.53
	B	25.34	−8.65	7.90	−10.58	−9.57	−15.40	−20.88	−27.37
20–40	N	−18.56	13.15	16.16	10.14	32.31	4.36	13.71	14.90
	**G**	**−25.10**	**30.30**	**21.14**	**9.50**	**45.85**	**23.70**	**42.63**	**−4.35**
	M	16.78	51.47	9.66	−7.28	14.10	8.02	34.03	−33.09
	B	−26.11	−12.95	−10.25	−6.34	9.18	−46.57	−9.07	−10.52
40–60	N	−6.18	13.64	11.34	6.91	16.58	12.51	9.99	−1.56
	**G**	**23.75**	**35.02**	**13.64**	**−18.67**	**33.10**	**13.70**	**25.14**	**−1.88**
	M	37.88	16.15	18.22	26.13	7.17	−3.52	−0.07	17.77
	B	−23.07	−10.27	−3.49	4.95	−26.82	−26.24	−15.46	14.15

Description: disturbance intensity (DI) (N, G, M, and B are, respectively, no disturbance (the number of effective burrows was 0), mild disturbance (the number of effective burrows was 1–3), moderate disturbance (3–6 effective burrows), and severe disturbance (the effective number of burrows was >6)). The enrichment ratio (E%) with a value >0 indicates nutrient enrichment and a value <0 imply nutrient depletion. TOC: soil organic carbon, TN: soil total nitrogen, TP: soil total phosphorus, TK: total potassium, AN: nitrogen, AP: available phosphorus, AK: available potassium and, TS: total salt. The values in bold in Table 1 have no special definition.

### 3.2 Correlation analysis between *R. opimus* disturbance and soil nutrients

The Pearson correlation analysis results showed that soil nutrient contents were significantly correlated with *R. opimus* DIs (*p* < .05; Table 2). Specifically, DIs were significantly and positively correlated with soil TP and AN contents (*p* < .05). Moreover, it demonstrated a highly significant and negative correlation with soil AP and TS contents in R soil (*p* < .01) but a significant and positive correlation with soil TN, TK, AK, TP, and AN contents in S soil (*p* < .01). In R and S soils, TS content was significantly and negatively correlated with TOC, TP, TN, and AN contents (*p* < .01); AK content was significantly and positively correlated with TP, AN, and AP contents (*p* < .01); AN content was significantly and positively correlated with TP contents (*p* < .05); and TN content was significantly and positively correlated with TOC content (*p* < .01).

**TABLE 2:** Pearson correlation coefficients among soil nutrient in rhizosphere (below the diagonal) and nonrhizosphere (upper the diagonal) soil of *Haloxylon ammodendron* in different disturbance intensity

Variables	TOC (g/kg)	TN (g/kg)	TP (g/kg)	TK (g/kg)	AN (mg/kg)	AP (mg/kg)	AK (mg/kg)	TS (mg/g)	DI
TOC (g/kg)		0.720**	0.1	−0.038	0.111	0.284	0.529**	−0.389*	−0.058
TN (g/kg)	0.711**		0.299	0.125	0.221	0.008	0.392*	−0.542**	0.349*
TP (g/kg)	−0.037	−0.031		0.584**	0.712**	0.17	0.525**	−0.397*	0.753**
TK (g/kg)	−0.3	−0.474**	−0.029		0.541**	0.021	0.212	−0.268	0.386*
AN (mg/kg)	0.325	0.385*	0.345*	0.031		0.226	0.527**	−0.632**	0.834**
AP (mg/kg)	0.113	−0.08	0.234	0.165	−0.047		0.634**	0.157	0.098
AK (mg/kg)	0.259	0.304	0.337*	0.179	0.563**	0.535**		−0.269	0.332*
TS (mg/g)	−0.556**	−0.505**	−0.356*	0.296	−0.765**	0.257	−0.179		−0.530**
DI	−0.004	0.282	0.340*	−0.036	0.364*	−0.485**	−0.236	−0.518**	

Description: The values are the Pearson correlation coefficients. The correlation coefficient r of Pearson is between −1 and 1, *r* < 0 is negative correlation, and *r* > 0 is positive correlation. * represent significances at *p* < .05; ** represents significances at *p* < .01.

### 3.3 Effects of *R. opimus* disturbance on *H. ammodendron* nutrient components

**FIGURE 4:**
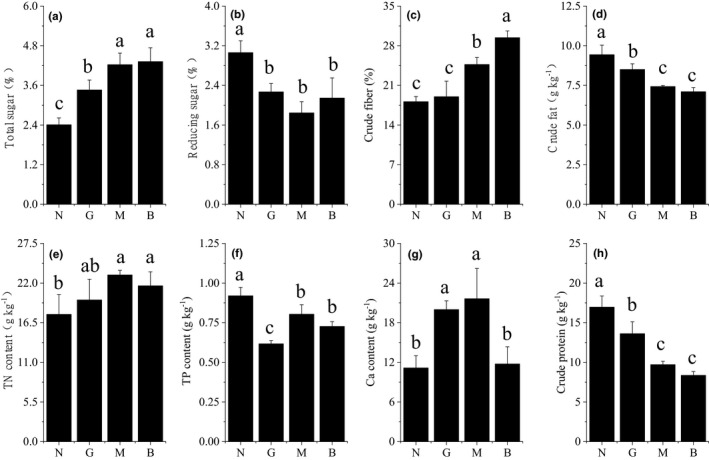
FIGURE Effects of different disturbance intensity on nutrient composition of branches of *Haloxylon ammodendron*. Description: Ordinate is the content of soluble sugar (STS) (A), reductive sugar (RS) (B), crude fiber (CF) (C), crude fat (CFT) (D), total nitrogen (TNC) (E), total phosphorus (TPC) (F), calcium (Ca) (G), and crude protein (CP) (H). Abscissa is the group name that has identical meanings as described in Figure 2

The results showed that *R. opimus* disturbance significantly affected the nutrient content in the collected *H. ammodendron* branches (Figure 4). Specifically, as *R. opimus* DI increased, the contents of STS (Figure 4A), CF (Figure 4C), and TNC (Figure 4E) in the collected *H. ammodendron* branches all showed a significant increasing trend, whereas the contents of crude fat (CFT) (Figure 4D) and crude protein (CP; Figure 4H) showed a significant decreasing trend (*p* < .05). Additionally, the content of reducing sugar (RS) was higher in N, while all other treatments recorded similar reducing sugar content (Figure 4B). The content of TPC was higher in N, and the least was recorded in G (Figure 4F). In particular, TPC content under G was significantly lower than that under other DIs (*p* < .05). Moreover, Ca content was significantly higher in G and M and lower in N and B (Figure 4G).

### 3.4 Effects of *R. opimus* disturbance on the N/P ratio in the collected branches and R soils

**FIGURE 5:**
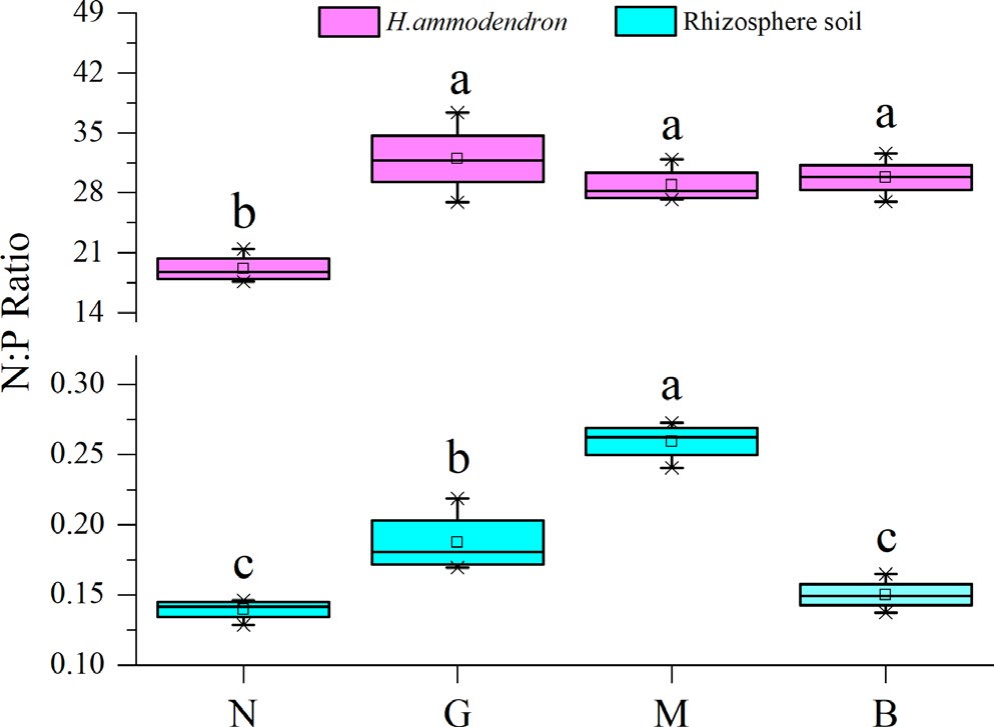
Effects of different disturbance intensity on N:P ratio in *Haloxylon ammodendron* and rhizosphere soil. Description: Ordinate is N:P ratio in *H. ammodendron* and rhizosphere soil; abscissa is the group name that has identical meanings as described in Figure 2

As shown in Figure 5, *R. opimus* disturbance affected N/P ratio in R soils (*p* < .05). Specifically, the mean N/P ratios under N, G, M, and B were, respectively, 19.16, 31.95, 28.89, and 29.78 in the collected *H. ammodendron* branches and 0.14, 0.19, 0.26, and 0.15 in R soils.

### 3.5 Relationship between the nutrients in the *H. ammodendron* branches and R soils

Nutrient contents in the collected *H. ammodendron* were significantly affected by *R. opimus* disturbance. As shown in Table 3, *R. opimus* DIs were significantly correlated with all the nutrients, except TPC and Ca, in the collected *H. ammodendron* branches (*p* < .05). In the terms of relationship between nutrient composition of collected *H. ammodendron* branches and R soils, STS content was significantly and positively correlated with TOC and AN content but significantly and negatively correlated with AP and TS (*p* < .05). In R soils, RS content was significantly and positively or negatively correlated with the contents of all nutrients, except TP and AK (*p* < .05). In particular, CF content was significantly and negatively correlated with AP and TS contents (*p* < .05). TNC content was significantly and positively correlated with TOC and TN contents but significantly and negatively correlated with AP and TS contents (*p* < .05). TPC content was significantly and negatively correlated with TOC, TP, AN, and AK contents but significantly and positively correlated with TS content (*p* < .05). Ca content was significantly and positively correlated with TOC, TN, and AN contents (*p* < .05). CP content was significantly and negatively correlated with TOC and AN contents but significantly and positively correlated with AP and TS contents (*p* < .05).

**TABLE 3:** Correlation analysis of rhizosphere soil and branches nutrients of *Haloxylon ammodendron* in different disturbance intensities

Variables	STS (%)	RS (%)	CF (%)	CFT (g/kg)	TNC (g/kg)	TPC (g/kg)	Ca (g/kg)	CP (g/kg)
TOC (g/kg)	0.767**	−0.784**	0.345	−0.659*	0.616*	−0.626*	0.721**	−0.643*
TN (g/kg)	0.481	−0.686*	0.195	−0.411	0.593*	−0.247	0.723**	−0.440
TP (g/kg)	0.385	−0.349	0.421	−0.427	0.229	−0.735**	−0.015	−0.375
TK (g/kg)	−0.48	0.644*	−0.251	0.383	−0.568	0.018	−0.390	0.411
AN (mg/kg)	0.716**	−0.781**	0.399	−0.638*	0.493	−0.868**	0.588*	−0.630*
AP (mg/kg)	−0.646*	0.682*	−0.579*	0.670*	−0.634*	−0.100	−0.461	0.678*
AK (mg/kg)	−0.272	0.197	−0.439	0.360	−0.414	−0.655*	0.072	0.352
TS (mg/g)	−0.858**	0.907**	−0.581*	0.800**	−0.614*	0.710**	−0.561	0.806**
DI	0.849**	−0.586*	0.941**	−0.883**	0.582*	−0.267	−0.119	−0.914**

Description: The values are the Pearson correlation coefficients. The correlation coefficient r of Pearson is between −1 and 1, *r* < 0 is negative correlation, *r* > 0 is positive correlation. * represent significances at *p* < .05; ** represent significances at *p* < .01.

Furthermore, nutrient composition in the collected branches of *H. ammodendron* significantly correlated (*p* < .05; Table 4). Specifically, STS content was significantly and negatively correlated with RS, CFT, and CP contents but significantly and positively correlated with CF and TNC contents and RNP (N/P ratio) (*p* < .05). RS content was significantly and positively correlated with TK, AP, AK, and TS but significantly and negatively correlated with TOC, TN, TP, AN, and DI (*p* < .05). CF content was significantly and negatively correlated with CFT and CP but significantly and positively correlated with TNC content (*p* < .05). CFT content was significantly and negatively correlated with TNC content and RNP (N/P ratio) but significantly and positively correlated with CP content (*p* < .05). TNC content was significantly and negatively correlated with CP content but significantly and positively correlated with RNP (N/P ratio) (*p* < .05). CP content was significantly and negatively correlated with RNP (N/P ratio) (*p* < .05).

**TABLE 4:** Correlation analysis between nutrients in the assimilation branches of *Haloxylon ammodendron*

Variables	STS (%)	RS (%)	CF (%)	CFT(g/kg)	TNC (g/kg)	TPC (g/kg)	Ca (g/kg)	CP (g/kg)
RS (%)	−0.809**							
CF (%)	0.738**	−0.552*						
CFT(g/kg)	−0.916**	0.740**	−.873**					
TNC (g/kg)	0.697**	−0.652**	.632**	−0.691**				
TPC (g/kg)	−0.468	0.516*	−0.161	0.401	−0.177			
Ca (g/kg)	0.291	−0.544*	−0.075	−0.268	0.401	−0.374		
CP (g/kg)	−0.941**	0.769**	−0.859**	0.929**	−0.694**	0.357	−0.198	
RNP	0.662**	−0.693**	0.426	−0.620*	0.691**	−0.823**	0.488	−0.573*

Description: The values are the Pearson correlation coefficients. The correlation coefficient r of Pearson is between −1 and 1, *r* < 0 is negative correlation, *r* > 0 is positive correlation. * represents significances at *p* < .05; ** represents significances at *p* < .01.

### 3.6 Effects of environmental factors on nutrient composition in the *H. ammodendron* branches

**FIGURE 6:**
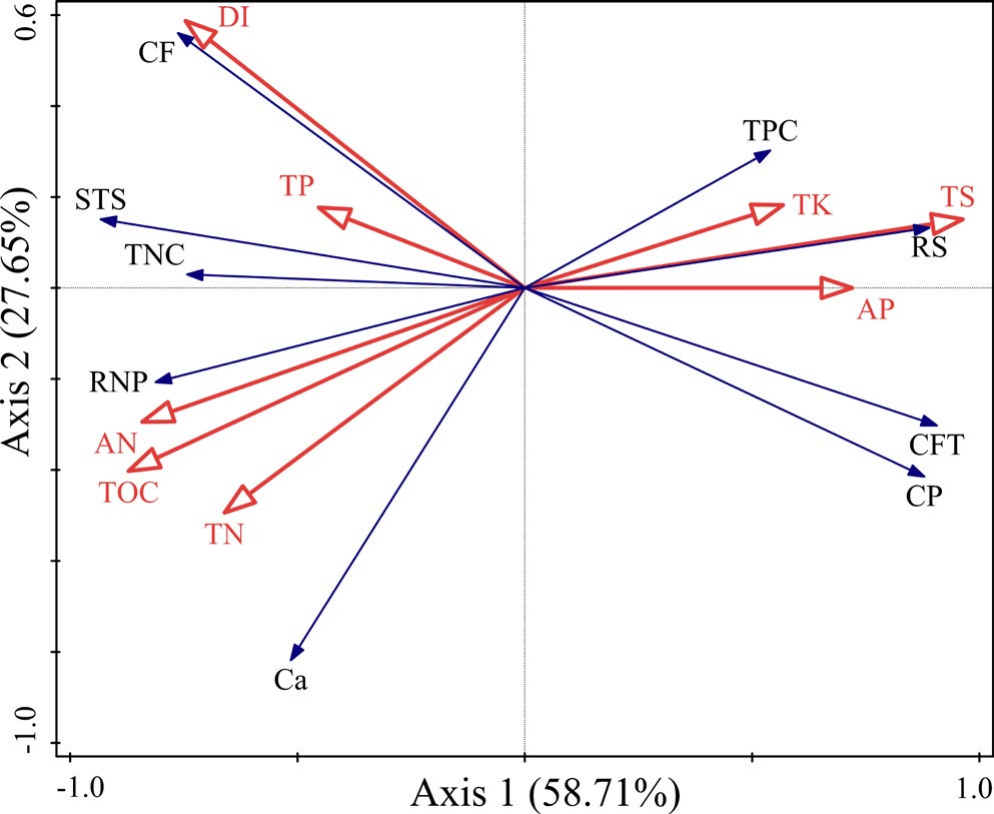
Redundancy analysis (RDA) for environmental factors. Description: Environmental factors are generally represented by arrows. The length of the arrow line represents the degree of correlation between a certain environmental factor and community and species distribution, and the longer the arrow, the greater the correlation. When the angle between the environmental factors is acute, it means that there is a positive correlation between the factors, an obtuse angle implies a negative correlation. Red and blue arrows represented rhizosphere soil factors and nutrient composition of *Haloxylon ammodendron* assimilative branches, respectively

The RDA results showed that environmental factors significantly affected the nutrient composition in the *H. ammodendron* branches (Figure 6), the total variance between environmental factors and nutrient composition in the collected branches was 5.72, and the explanatory variables accounted for 95.2% of the total variance. The first two principal component axes accounted for 86.07% of the total variance in the nutrient composition–environmental factor relationship; of them, the first and second ordination axes were responsible for 58.71% and 27.65% of the total variance, respectively.

**FIGURE 7:**
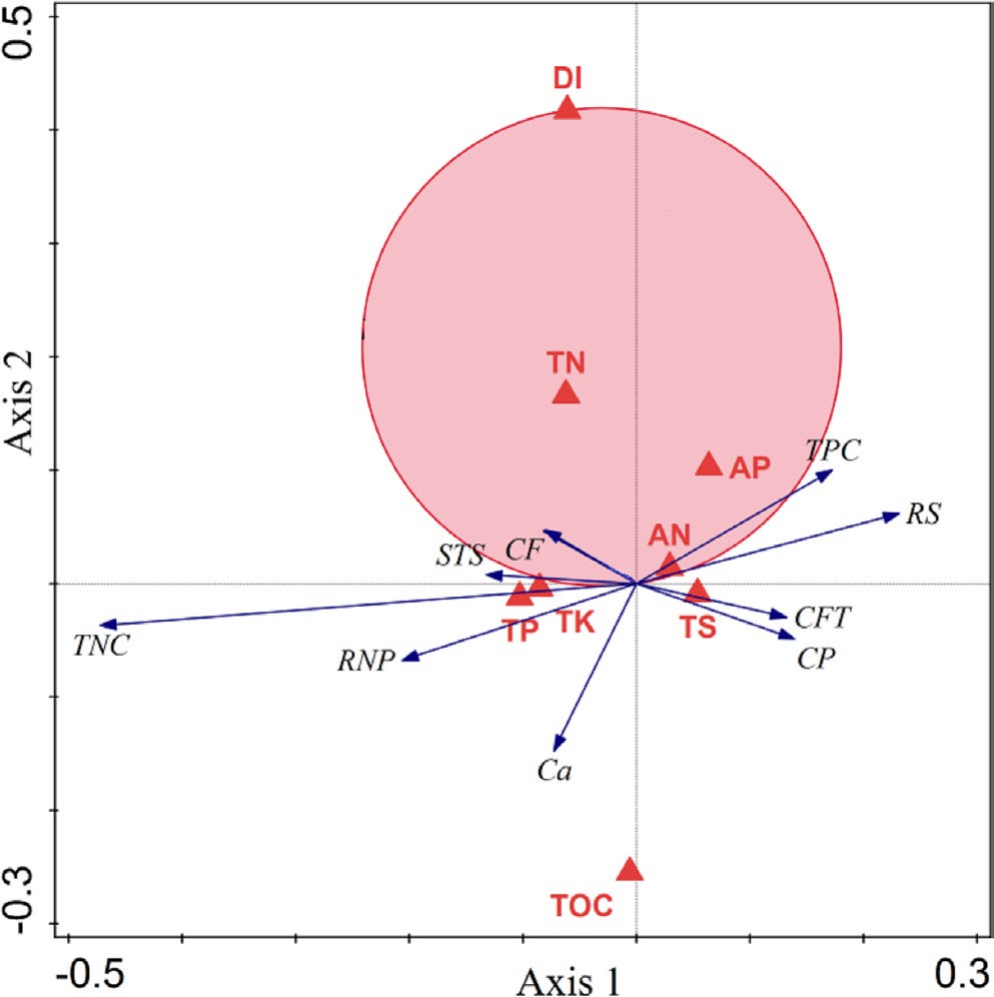
T‐value bioplot of disturbance intensity (DI) on the relationship between *Haloxylon ammodendron* and environmental variables. Description: Environmental factors are generally represented by arrows. The length of the arrow line represents the degree of correlation between a certain environmental factor and community and species distribution, and the longer the arrow, the greater the correlation. When the angle between the environmental factors is acute, it means that there is a positive correlation between the factors, and an obtuse angle implies a negative correlation

As shown in Figure 6, R soil TS contents and DIs were the major factor contributing to the differences in the functional traits of the branches of *H. ammodendron* (*p* = .002)—accounting for 86.36% of the total variance (Table 5)—followed by TOC and AN contents—accounting for 46.2% (*p* = .002) and 43.6% (*p* = .004) of the total variance, respectively (Table 5). These results were consistent with the analysis of T‐value bioplot of DIs on the relationship of *H. ammodendron* nutritional functional traits with environmental factors (Figure 7).

**TABLE 5:** The eigenvalues and significant test in RDA of the relationship between environmental factors and nutritional functional traits of *Haloxylon ammodendron*

Position	Variables	Explains (%)‐α	Explains (%)‐β	*F*‐value	*p*‐value
Rhizosphere	TS	53.8	58.71	16.3	.002
	DI	46.8	27.65	12.3	.002
	TOC	46.2	5.6	12	.002
	AN	43.6	2.8	10.8	.004
	TN	30.2	1.7	6.1	.008
	AP	30	2.2	6	.004
	TK	19	0.8	3.3	.036
	TP	18.2	0.54	3.1	.054

Description: Variable is the information of environmental factors. *p* value is the *p*‐value of significance test. *p* < .05 indicates statistical significance.

## 4 DISCUSSION

### 4.1 *R. opimus* disturbance significantly affected soil nutrient contents in R and S soils

Low nutrient content is a main feature of soil in desert areas. The “fertile island” effect, under the shrub of desert vegetation, is a crucial accumulation phenomenon observed under limited soil resources and the main mechanism in shrubs that facilitates their nutrient utilization and adaptation to barren environment in desert areas (Zhao et al., 2021; Tu et al., 2011; Yuefei et al., 2018).

In this study, we found that *R. opimus* disturbance significantly affected the processes of nutrient fixation, transfer, and redistribution and soil nutrient content in both R and S soils (Figures 2 and 3, Tables 1 and 2), thus promoting the formation of the fertile island effect. This result is consistent with the viewpoint of Szeman et al. (2008) that “animals are the transporters of desert soil nutrients under desert drought conditions.” Furthermore, during field investigations, we found a low amount of excrement inside the *R. opimus* burrows because most of the excrement were discharged at or transported out to the entrance of the burrows; this observation is consistent with that of Yang et al. (2009). This activity of cleaning burrows by the *R. opimus* made the soil mix with other litter, promoting the decomposition of soil organic matter. This somewhat explains the high TN and TOC contents in R and S soils at 0–20 cm depth (Figure 2B). Furthermore, in this study, *R. opimus* disturbance had impact on R and S soils; in particular, mild‐to‐moderate disturbance had positive effect on AN and AP contents in deeper layer soil (at 20–60 cm depth; Figures 2B and 3A,B), which greatly improved the nutrient adsorption capacity in *H. ammodendron* rhizosphere and the rhizosphere E increased (Figure 2A–C and Table 1). This was mainly due to the burrowing activities by *R. opimus*. We found that *R. opimus* burrows were mainly distributed in the soil layer at the depths of 20–40 cm depth. We found that *R. opimus* could store a considerable amount of food in the burrow area. This food is rich in nutrients and becomes the source of organic matter and other nutrients in deep soil layers (Herrera et al., 1999). In addition, the burrow structure can affect the soil ventilation and water conditions and shape the micro‐topography (Russell & Maclean, 2008), which is conducive to microbial decomposition and thus enriches deep soil nutrient content (Kuznetsova et al., 2013).

Although the severe disturbance by *R. opimus* increased TOC content in the soil surface layer at 0–20 cm depth, the taproot and lateral root of *H. ammodendron* were inevitably damaged by the over‐dense burrowing activities of *R. opimus*; this greatly weakened or even terminated the transport function of the *H. ammodendron* roots, thus greatly reducing their adsorption capacity, especially causing nutrient deficiency in the middle (20–40 cm) and deep (40–60 cm) soil layers (Figure 3A). The rhizosphere E reflects not only the degree of nutrient enrichment but also the strength of plant rhizosphere effect (Yang & Liu, 2015). In this study, the order of the strength of the *H. ammodendron* rhizosphere soil effect under different DIs was as follows: mild > moderate > no > severe (Table 1). This indicated that there is a threshold of *R. opimus* population disturbance intensity on *H. ammodendron* at rhizosphere soil. A DI within the threshold can enhance the benefit of soil nutrients on *H. ammodendron*, and excessive *R. opimus* feeding on *H. ammodendron* and its excessive burrow activity under a *H. ammodendron* canopy worsens the self‐healing ability of *H. ammodendron*, thus negatively affecting *H. ammodendron* vitality to a certain extent and weakening the adsorption capacity of the root system. This gradually leads to *H. ammodendron* population decline. In this study, moderate *R. opimus* disturbance (effective burrows number = 3–6) was the maximum disturbance *H. ammodendron* could withstand, whereas mild *R. opimus* disturbance (effective burrow number = 1–3) was the threshold for optimal response of *H. ammodendron* to *R. opimus* disturbance.

In recent years, the global climate change and human activities have led to an increase in the frequency of damage caused by rodents, mainly *R. opimus*, in desert areas. The mutually beneficial relationship between *H. ammodendron* and *R. opimus* is of great practical significance to effectively control rodent population density under the threshold value so as to protect desert plant diversity and ecological environment.

In the field investigation, we found that the high concentration of soil salinity is an important factor for *H. ammodendron* population decline—consistent with the report of Yang et al. The results demonstrated that *R. opimus* disturbance at different soil depths reduced R and S soil salt contents, and *R. opimus* DIs were significantly and negatively correlated with the soil total salt content (Table 2), possibly because soil density decreased and moisture changed during rodent burrowing activity. In addition, urine and feces was found to stimulate microbial activity and promote nitrogen‐fixing microorganism accumulation (Hawkins, 1996; Kuznetsova et al., 2013), thereby diluting soil salt concentration. At this level, *R. opimus* disturbance could aid *H. ammodendron* in avoiding soil salinization risk to a certain extent.

In the desert ecosystem, the fertile island effect causes the redistribution of soil resources (such as soil moisture, nutrient, and salt) in the whole system, resulting in continuous occurrence and development of “plundered” and barren soil patches. This promotes the invasion of herbaceous plants by shrubs, resulting in continuous degradation of herbaceous plants and acceleration of ecosystem desertification (Liu et al., 2006). In contrast, moderate *R. opimus* disturbance can somewhat supplement soil nutrients under *H. ammodendron*, reduce the predation of the surrounding soil nutrients by shrubs, and play a positive role in desert ecosystem restoration.

### 4.2 *R. opimus* disturbance was the main environmental factor affecting nutrients in the collected *H. ammodendron* branches

The selection of plants by herbivores results from the rich nutrients and palatability of the target plants, whereas the behavior and population dynamics of herbivores depend on plant nutrition and defense characteristics (Koussoroplis et al., 2019; Wetzel et al., 2016). In this study, we selected midsummer time (June) to study the correlation between *R. opimus* and *H. ammodendron*. This is because during this period, *R. opimus* stores large amounts of food in their burrows for the summer, when they need to hide from the heat. It is also the peak season for *H. ammodendron* growth.

We found that as *R. opimus* DIs increased so did Ca and CF contents (Figure 4G,C)—which may indicate a defense measure of *H. ammodendron* against *R. opimus* disturbance. Ca and cellulose are important constituents of plant cell walls (Rongpipi et al., 2019). Plants contain sufficient Ca and can easily form calluses; moreover, Ca participates along with TS in osmotic adjustment to increase stress resistance. Increases in cellulose content can reduce the feeding frequency of herbivores to food, which may be due to the overcompensating response of *H. ammodendron* in nutrition metabolism caused by *R. opimus* feeding. Alternatively, it may be a defensive response of *H. ammodendron* to *R. opimus* through nutrient composition adjustments. At high DIs, Ca content suddenly dropped (Figure 4G), possibly indicating that the DI has exceeded the threshold for *H. ammodendron*. This resulted in a decrease in its vitality and cell transportation and adsorption capacities. A decrease in CP content may be due to the increased Proline (Pro) decomposition. This is a common physiological index of stress resistance because Proline (Pro) participates in osmotic regulation and increases plant resistance during *R. opimus* feeding. TN content in the collected *H. ammodendron* branches was positively correlated with soil TN content and *R. opimus* DIs. Therefore, TN content in the collected *H. ammodendron* branches must have increased because of *R. opimus* disturbance so as to supplement soil nitrogen sources.

When faced with plant nutrient variability, herbivores' physiological activities are consistently restricted, resulting in reduced nutritional performance (Wetzel et al., 2016). In this study, as *R. opimus* DI increases, the CP, CFT, and RS required by *R. opimus* in the collected *H. ammodendron* branches decreased and the contents of CF disliked by *R. opimus* increased gradually. This, these nutrients correlates with *R. opimus* DIs (Table 3, Figure 5)—possibly representing the “nutrition target transfer” defense strategy employed by plants against herbivores. In summary, the nutrient content changes in the collected *H. ammodendron* branches represent not only a physiological response to *R. opimus* disturbance but also a defense strategy against *R. opimus*.

The plant N/P ratio is an effective tool to indicate the nutrient limitation of plants in the study area (Ien & Moen, 2009). Zhang et al. (2004) proposed that the N/P ratio threshold can explain the N and P limitation in the study area: The growth of a species may be limited by N, P, and both N and P contents when the N/P ratio is <21, >23, and 21–23, respectively. Our current results showed that as *R. opimus* DIs increased, *H. ammodendron* growth in the study area gradually changed from N restriction (N/P = 19.16) to P restriction (N/P > 23; Figure 5). Moreover, soil TN content was significantly and positively correlated with *R. opimus* DIs, indicating that the nitrogen accumulation effect of *H. ammodendron* rhizosphere is strong under mild‐to‐moderate *R. opimus* DIs (E = 33–61%; Tables 1 and 2). Thus, moderate *R. opimus* disturbance somewhat alleviated the pressure of nitrogen deficiency in the desert ecosystem and supplemented a certain amount of nitrogen as a source.

## 5 CONCLUSIONS

The results of this study demonstrated the variations in *H. ammodendron* and soil nutrient contents caused by *R. opimus* different disturbance levels, thus providing new insights into rodent prevention and control and sustainable development of the ecosystem in the desert areas. The maximum *R. opimus* DI that *H. ammodendron* can withstand was noted to be moderate. Moreover, mild *R. opimus* DI was the threshold for optimal *H. ammodendron* response, which was the most beneficial to *H. ammodendron* growth and development. Furthermore, *R. opimus* disturbance was found to facilitate nutrient enrichment in the deep layer soil (at 20–60 cm depth). Moreover, the results indicated that *R. opimus* disturbance was found to be the main environmental factor affecting the nutritional function traits of *H. ammodendron*, and as *R. opimus* DI increased, *H. ammodendron* plant nutrient content increased significantly—which may represent both plant physiological resistance regulation and “nutrient target transfer” defense strategy against herbivores. In general, moderate *R. opimus* disturbance was noted to alleviate the pressure of nutrient deficiency in a desert ecosystem to a certain extent, supplement certain nutrient sources, and enhance the rhizosphere effect of *H. ammodendron*—which plays a positive role in desert ecosystem restoration.

## CONFLICT OF INTEREST

The authors declare no conflict of interest.

## AUTHOR CONTRIBUTIONS

**Wenqin Zhao:** Data curation (equal); Investigation (equal); Methodology (equal); Writing‐original draft (equal). **Hanli Dang:** Formal analysis (equal); Methodology (equal); Resources (equal); Writing‐original draft (equal). **Tao Zhang:** Data curation (supporting); Formal analysis (supporting); Investigation (supporting); Methodology (supporting). **Jianrui Dong:** Formal analysis (supporting); Investigation (supporting); Methodology (supporting). **Hongwei Chen:** Investigation (supporting); Methodology (supporting); Software (supporting). **Wenjie Xiang:** Investigation (supporting); Resources (supporting); Software (supporting).

### OPEN RESEARCH BADGES

This article has earned an Preregistered Badge for making publicly available the digitally‐shareable data necessary to reproduce the reported results. The data is available at https://doi.org/10.6084/m9.figshare.14844633.

## Data Availability

The data used in this manuscript are available at Figshare: https://doi.org/10.6084/m9.figshare.14844633.
